# Therapeutic Use of mTOR Inhibitors in Renal Diseases: Advances, Drawbacks, and Challenges

**DOI:** 10.1155/2018/3693625

**Published:** 2018-10-29

**Authors:** Sofia D. Viana, Flávio Reis, Rui Alves

**Affiliations:** ^1^Laboratory of Pharmacology & Experimental Therapeutics, Coimbra Institute for Clinical and Biomedical Research (iCBR), Faculty of Medicine, CNC.IBILI Consortium & CIBB Consortium, University of Coimbra, 3000-548 Coimbra, Portugal; ^2^Polytechnic Institute of Coimbra, ESTESC-Coimbra Health School, Pharmacy, 3046-854 Coimbra, Portugal; ^3^Nephrology Department, Coimbra University Hospital Center & University Clinic of Nephrology, Faculty of Medicine, University of Coimbra, 3000-075 Coimbra, Portugal

## Abstract

The mammalian (or mechanistic) target of rapamycin (mTOR) pathway has a key role in the regulation of a variety of biological processes pivotal for cellular life, aging, and death. Impaired activity of mTOR complexes (mTORC1/mTORC2), particularly mTORC1 overactivation, has been implicated in a plethora of age-related disorders, including human renal diseases. Since the discovery of rapamycin (or sirolimus), more than four decades ago, advances in our understanding of how mTOR participates in renal physiological and pathological mechanisms have grown exponentially, due to both preclinical studies in animal models with genetic modification of some mTOR components as well as due to evidence coming from the clinical experience. The main clinical indication of rapamycin is as immunosuppressive therapy for the prevention of allograft rejection, namely, in renal transplantation. However, considering the central participation of mTOR in the pathogenesis of other renal disorders, the use of rapamycin and its analogs meanwhile developed (rapalogues) everolimus and temsirolimus has been viewed as a promising pharmacological strategy. This article critically reviews the use of mTOR inhibitors in renal diseases. Firstly, we briefly overview the mTOR components and signaling as well as the pharmacological armamentarium targeting the mTOR pathway currently available or in the research and development stages. Thereafter, we revisit the mTOR pathway in renal physiology to conclude with the advances, drawbacks, and challenges regarding the use of mTOR inhibitors, in a translational perspective, in four classes of renal diseases: kidney transplantation, polycystic kidney diseases, renal carcinomas, and diabetic nephropathy.

## 1. Introduction

The mechanistic (formerly mammalian) target of rapamykinase, was discovered almost simultaneously by three independent groups in the mid-1990s and coined as rapamycin and FK506-binding protein-12 (FKBP-12) target 1 (RAFT1), FKBP–rapamycin-associated protein (FRAP), and mTOR [1–3]. These names reflected the fact that mTOR was identified as the target of rapamycin (etymol.: Rapa- (Rapa Nui = Easter Island), -mycin (related to the antifungal properties)), which is a natural antibiotic macrolide firstly isolated from bacterium (Streptomyces hygroscopicus) extracts found on Easter Island soil samples [4].

mTOR is a member of the phosphatidylinositol 3-kinase-related kinase (PIKK) family, which is one of the key players of cellular metabolism that is coupled with nutrient availability, energy, and homeostasis [5, 6]. It plays a prominent role as a molecular sensor of gene transcription and protein synthesis, tissue regeneration and repair, immunity, oxidative stress, and cell proliferation/cell death (e.g., autophagy and apoptosis) upon environmental and cellular cues (nutrients (e.g., glucose, amino acids, and fatty acids), growth factors (e.g., insulin-like growth factor-1, IGF-1; vascular endothelial growth factor, VEGF), hormones (e.g., insulin), and cytokines) [7–9]. Given the ubiquitous distribution of mTOR in distinct cell types throughout the body, mTOR pathway control several anabolic and catabolic processes in distinct organs/tissues including (but not restricted) the liver, lymphocytes, white and brown adipose tissue, skeletal muscle, brain, heart, and kidney [8]. Hence, impaired mTOR activity has been associated in widespread human diseases, including cancer, type 2 diabetes, cardiovascular pathology, and neurodegeneration as well as during aging [10–12].

Notably, accumulated evidence suggests mTOR signaling deregulation as a central player in the pathophysiology of distinct kidney diseases. Herein, we will critically discuss the advances, drawbacks, and future challenges of mTOR pharmacological inhibition in distinct renal conditions and in a bench-to-bedside perspective.

## 2. Overview of mTOR Components and Signaling Pathways

mTOR is a 289 kDa protein kinase encoded in humans by the *MTOR* gene (1p36.2). It interacts with several proteins to form two evolutionary conserved complexes among eukaryotes—mTORC1 and mTORC2. There are two common proteins shared by mTORC1/mTORC2 multimeric complexes: the positive regulator mLST8 (mammalian lethal with Sec13 protein8, also known as G*β*L) and the negative regulator Deptor (DEP domain-containing mTOR-interacting protein). Yet, there are unique proteins coupled to each complex: mTORC1 is associated with raptor (regulatory-associated protein of mTOR), fundamental for mTORC1 stability and a positive regulator of downstream effectors recruitment and with PRAS40 (proline-rich Akt substrate), a protein which blocks mTORC1 activity; mTORC2 is coupled with mSIN-1 (mammalian stress-activated protein kinase-interacting protein), PROTOR 1/2, and Rictor (rapamycin-insensitive companion of mTOR), a scaffold protein that displays chief roles for mTORC2 assembly, stability, and substrate recognition (e.g., Akt and SGK1) [7, 11].

The mTOR-containing complexes also differ in terms of upstream modulators, substrate specificity, functional outputs, and sensitivity to rapamycin [13]. mTORC1 broadly senses nutrients, growth factors, mitogens, and stress signals, thus being generally associated with cell growth by regulating important cellular processes, including the translation of mRNAs into the synthesis of key proteins for proliferation, lipid synthesis, mitochondrial biogenesis, and autophagy [14, 15]. Examples of mTORC1 downstream effectors are the lipin 1/SREBP (sterol regulatory element-binding proteins), the p70S6 kinases (S6K1 and S6K2), and the EIF4EBP1 (eukaryotic translation initiation factor 4E-binding protein 1) [16, 17]. In contrast to mTORC1, the control of mTORC2 by upstream modulators and downstream effector proteins is not as well understood, even though insulin and related pathways have been suggested as the main activators [11]. Nevertheless, plasma membrane localization as well as ribosome-binding through insulin-stimulated phosphatidylinositol 3-kinase (PI3K) signaling seem to have a chief role in mTORC2 regulation [18, 19]. Phosphorylation of protein kinase B (Akt) and other AGC-family kinases (e.g., serum- and glucocorticoid-induced protein kinase 1, SGK1; protein kinases C, PKC) has been linked with mTORC2 activation, with important consequences on cell survival, cytoskeleton organization, and cycle progression [14, 20, 21]. Interestingly, Akt appears to have a complex dual role on mTOR, being both an (i) upstream regulator of mTORC1 (indirect activation through phosphorylation and inactivation of TSC1/TSC2 complex, who constitutively suppress mTORC1 activity through Rheb GTPase inhibition) and a (ii) downstream target of mTORC2 [10, 22]. The activity of the two complexes is finely and mutually tuned through some feedback circuits promoted not only by upstream regulators of mTORC1 (e.g., Akt) but also by other downstream effectors of mTORC1, such as the p70S6K1 [23–25]. For instance, p70S6K1 phosphorylates mSIN-1 at both Thr86 and Thr389 residues and dissociates mSIN-1 from mTORC2, thus providing a negative feedback mechanism downstream of mTORC1. The loss of such reciprocal mechanistic feedback loops is observed in some mutational loss-of-function in mTOR key components, as in the case of the R81T Sin1 mutation identified in ovarian cancer patients, highlighting their clinical relevance [25]. Hence, impairments of constitutive feedback mechanisms and unexpected mTOR hyperactivation are particularly relevant when mTOR signaling modulation is envisaged. In this regard, the fact that PI3K/Akt/mTOR pathway critically regulates a plethora of physiological processes that become deregulated in a wide spectrum of pathologic conditions prompt the design of several pharmacological agents that target distinct components of this signaling cascade, as outlined in Section 3 [22, 26–28].

Finally, the sensitivity to rapamycin is another important feature that distinguishes mTORC1 and mTORC2 complexes. Rapamycin does not directly inhibit the catalytic (kinase) activity of mTOR; instead, it binds to the immunophilin FKBP12 (FK506-binding protein of 12 kDa), which is a protein that couples with mTOR FKBP-rapamycin-binding domain (FRB). Even though the FRB domain is present in both mTORC1/2 complexes, it is only exposed in mTORC1, as Rictor blocks the access of FKBP12-rapamycin complex to FRB domain in mTORC2 [25, 29]. Hence, mTORC2 is relatively resistant to the effects of rapamycin both *in vitro* and *in vivo*, although this phenomenon can be disrupted by chronic treatments [13, 30, 31].

## 3. Pharmacological Advances and Challenges within mTOR Inhibition

The recent breathtaking advances in up- and downstream targets of mTOR, reciprocal feedback mechanistic loops, and mutational loss-of-function in mTOR key components (e.g., TSC1/2, PIK3CA, and Akt), the most common cause of mTOR signaling hyperactivity, provided new rationales for translating the mTOR basic science to the clinic. In fact, pharmaceutical companies have discovered impressive arrays of small molecules targeting PI3K/Akt/mTOR cascade elements which are currently undergoing evaluation in preclinical and clinical studies mainly in cancer and transplantation, even though mTOR inhibitors are being also considered for other pathological conditions such as rheumatoid arthritis, atherosclerosis and a wide spectrum of neurologic disorders where aberrant mTOR pathway activity is consistently observed [22, 27, 28, 32]. Herein, it will be focused on the different classes of mTOR inhibitors currently undergoing preclinical/clinical studies aimed at providing new pharmacological agents with increased efficacy and a lower side effect profile.

### 3.1. Allosteric mTOR Inhibitors: Rapamycin/Rapalogues

**Figure 1:**
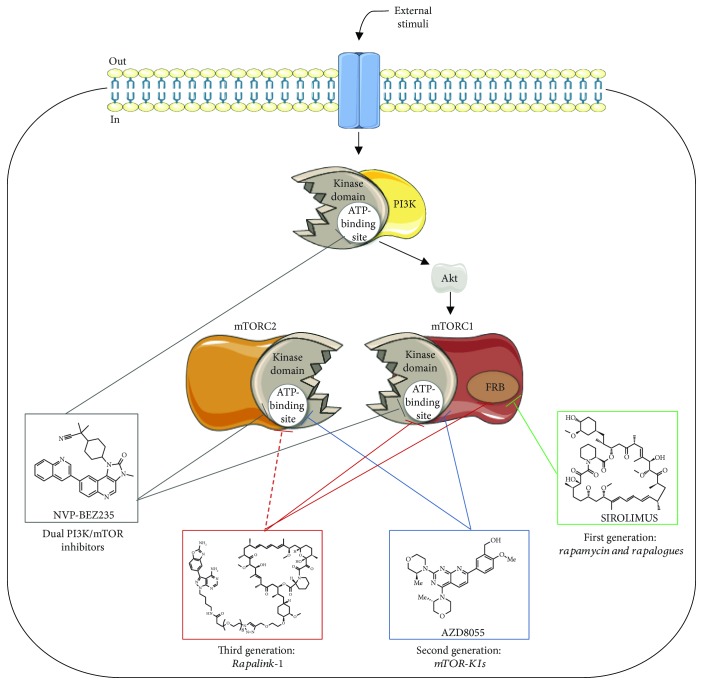
Mechanisms of action of three distinct generations of mTOR inhibitors and dual PI3K/mTOR inhibitors. The first generation of mTOR inhibitors (sirolimus chemical structure selected as an example) interacts with FRB domain of mTORC1 and partially inhibits mTOR downstream signaling events. Dual PI3K/mTOR inhibitors indiscriminately bind to the ATP-binding site of mTOR and PI3K catalytic domains, thus blocking the activity of both kinases (NVP-BEZ235 chemical structure selected as an example). The second generation of mTOR inhibitors act as ATP analogs and compete with ATP only in mTORC1/2 catalytic domains without substantial effect on PI3K (AZD8055 chemical structure selected as an example). Finally, the third generation of mTOR inhibitors combines a mTOR kinase inhibitor with rapamycin within the same molecule, which allows compounds to interact with the FRB domain and also to reach mTORC1 kinase domain, acting as an ATP-competitive inhibitor (Rapalink-1 chemical structure selected as an example). Dashed arrow represents the ability of Rapalink-1 to inhibit mTORC2 kinase activity, even though the precise molecular mechanism remains to be fully addressed (mTOR, mammalian target of rapamycin; PI3K, phosphatidylinositol 3-kinase; Akt, protein kinase B; FRB, FKBP-rapamycin-binding domain). Elements of the scheme were drawn using the website https://smart.servier.com/.

Rapamycin (or sirolimus), a macrocyclic lactone, was initially described as an antibiotic agent. Nevertheless, this molecule also exhibits immunosuppressant, cytostatic, antiangiogenic, and antiproliferative properties, expanding the clinical applications to transplantation and oncology fields [10]. Rapamycin acts as an allosteric inhibitor of mTORC1, which, together with FKBP12, interacts with the FRB domain of mTORC1 blocking some of the functions of this complex (see Figure 1). The data suggest that rapamycin impairs mTORC1 activity mainly by preventing the association and phosphorylation of substrates into the kinase complex [33, 34]. However, not all mTORC1 downstream targets are equally inhibited by rapamycin, with potency varying for weak versus strong substrates [25]. Moreover, and even though rapamycin does not interact with mTORC2, some studies have shown that this molecule is able to indirectly modify mTORC2 complex in a dose-, time-, and cell-type dependent manner, probably by preventing mTOR molecules from the interaction with mTORC2-specific partner protein Rictor [3, 31, 35].

The fact that rapamycin has limited bioavailability led to the development of semisynthetic analogs, named rapalogues, with superior aqueous solubility and improved pharmacokinetic properties. Examples of this first-generation of mTOR inhibitors are temsirolimus (CCI-779), everolimus (RAD001), and ridaforolimus/deforolimus (MK-8669/AP23573) who share a central macrolide chemical structure yet differ in the functional groups added at C40 that significantly alter bioavailability, half-life, and administration routes (oral versus intravenous) [22]. In contrast with everolimus and ridaforolimus, temsirolimus is a prodrug that requires removal of the dihydroxymethyl propionic acid ester group after administration, becoming sirolimus in its active form [36]. Rapalogues exhibit a safe toxicity profile, with side effects such as skin rashes and mucositis being dose-dependent. Other symptoms described are fatigue, anemia, neutropenia, and metabolic disorders such as hypertriglyceridemia, hypercholesterolemia, and hyperglycemia [22]. In this regard, it should be highlighted that rapamycin prevented insulin-mediated suppression of hepatic gluconeogenesis and impaired *in vitro* basal and insulin-stimulated glucose uptake in adipocytes from human donors [37, 38]. Temsirolimus and sirolimus are also associated with pulmonary toxicity, being interstitial lung disease, risk of secondary lymphoma, and reactivation of latent infections rare side effects [39].

Since mTORC1 and mTORC2 control events intimately related to cell growth and survival, rapalogues have been extensively studied in the oncology field, with several works conducted to analyze the effectiveness of these class of molecules alone and/or in combination with standard chemotherapy in the treatment of several types of cancers [26]. Although clinically promising, the results of such studies are quite disappointing, and some putative explanations have been hypothesized. Rapalogues have some serious drawbacks in terms of the desired molecular effects, and the efficacy may be partially limited by their drug action (cytostatic rather than cytotoxic). Moreover, as rapamycin and rapalogues act only on mTORC1, treatment with any of the molecules can elicit long-term feedback loops deregulation in mTOR network, therefore leading to aberrant activity of compensatory prosurvival pathways, including the PI3K/Akt signaling network itself. This phenomenon can seriously compromise the anticancer efficacy as well as the acquisition of chemoresistant phenotypes [22, 23, 25]. Since mTOR is a member of PIKK-related family sharing a high degree of similarity/sequence homology within the catalytic domain with PI3K, the next logical approach was the development of ATP-competitive dual PI3K/mTOR inhibitors.

### 3.2. Dual PI3K/mTOR Inhibitors

As highlighted above, rapamycin and rapalogues are incomplete inhibitors of mTORC1 and elicit feedback activation of PI3K/Akt mitogenic pathways. This argues for a theoretical therapeutic advantage of dual PI3K/mTOR inhibition in terms of better efficacy and less likelihood to induce drug resistance. These new agents are a class of catalytic ATP competitive inhibitors that exert their effects by binding indiscriminately to the ATP-binding site on both mTORC1/2 and PI3K catalytic domains (see Figure 1), which are two crucial signaling hubs [26, 40]. The prototype molecule in this class is the pyridofuropyrimidine PI-103, even though it was never translated into the clinic mainly because of its rapid *in vivo* metabolism [41, 42]. Over the next few years, other dual PI3K/mTOR inhibitors were discovered and advanced into the clinical evaluation (phase 1 and 2 trials), namely, the imidazoquinoline derivative NVP-BEZ235 (dactolisib), GDC-0980 (apitolisib), and PKI-587 (gedatolisib) [26, 40]. Although the appealing prospects of simultaneously targeting PI3K/mTOR, clinical studies have revealed a limited efficacy and important toxicity concerns (e.g., nausea, diarrhea, vomiting, decreased appetite, hyperglycemia, mucositis, cutaneous rash, elevated liver enzyme levels, renal failure, and hypertension). Moreover, it was proposed that dual PI3K/mTOR inhibitors suppressed a yet unidentified negative feedback loop mediated by mTORC2, which could partially explain the *in vitro* resistance and limited efficacy *in vivo* [25, 43].

### 3.3. ATP Competitive Inhibitors: mTOR Kinase Inhibitors (TOR-KIs)

More recently, a second-generation of pharmacological mTOR inhibitors have been developed. In contrast to the rapamycin analogs, these molecules exert their effects by directly blocking the ATP catalytic site that is integral to both mTOR complexes (see Figure 1), resulting in widespread inhibition of the mTOR signal [36, 44, 45]. These agents exhibit a much lower half-maximal inhibitory concentration (IC_50_) against mTOR activity than PI3K [26]. Hence, they are more discerning in their function: the main target is the mTORC1/2 catalytic domain without substantial effect on PI3K, with an expected reduction of toxicological events associated with dual PI3K/mTOR inhibitors [43]. Remarkably, mTOR kinase inhibitors (TOR-KIs) were effective antiproliferators in cell models displaying insensitivity to the first-generation of mTOR inhibitors [46, 47]. The first such compound was PP242, with numerous other TOR-KIs subsequently discovered, including Torin 1 and its sister Torin 2, AZD8055, TAK-228, and CC-223, some of them currently undergoing phase 1/2 clinical evaluation in neoplastic disorders [25, 26]. Nevertheless, mechanisms of resistance were already reported for these second generation of compounds, highlighting the many adaptive skills of PI3K/Akt/mTOR network upon modulation of any key component [25]. Among several reasons that may concurrently explain such discouraging results are feedback loops dysregulation as well as a wide range of mTOR mutations responsible for the increased catalytic activity of both mTORC1/2 complexes, rather than a direct active-site mutation interfering with drug binding [48–50].

### 3.4. New Generation: RapaLink-1

Considering the poor efficacy, resistance mechanisms, and severe side effects described for the class of drugs previously mentioned, an attempt to develop a third generation of mTOR inhibitors have been recently outlined. Through exploitation of both ATP- and FRB-binding sites of mTOR, the new molecule RapaLink-1 combine the high affinity of rapamycin for mTORC1 with the effective kinase inhibition of the TOR-KI MLN0128, which is a highly selective structural analog of PP242 that is currently in clinical trials [50]. The linker portion between these two molecules—a polyethylene glycol unit—does not disrupt rapamycin binding to FKBP12 or the FRB domain of mTOR, thus leveraging the high selectivity and affinity of rapamycin for mTORC1 and the “deliver” of MLN0128 to the ATP site of mTORC1 [50, 51]. Notably, RapaLink-1 was effective in the inhibition of both mTORC1 and mTORC2 downstream targets (mTORC1 (S6K, 4EBP1) and mTORC2 (Akt)) at doses between 1 and 3 nM, suggesting that it is also able to suppress the catalytic activity of both mTORC2 components through direct or indirect mechanisms that remain to be elucidated (see Figure 1). This drug was found effective in reversing resistance of breast cancer due to mTOR FRB or kinase domain mutations [50]. Despite its size, Rapalink-1 can cross blood-brain barrier and has shown increased efficacy in a glioblastoma cell model as well as in a genetically engineered *in vivo* model of brain cancer, when compared with earlier mTOR inhibitors [52]. Moreover, this compound did not display significant toxicity events when given intraperitoneally in mice and was also recently suggested as a possible new alternative to treat and prevent the development of alcohol use disorder (AUD) [53]. Overall, Rapalink-1 shows an appealing potency profile compared with earlier mTOR inhibitors which encourage next clinical evaluation. Nevertheless, further preclinical studies aimed at establishing whether Rapalink-1 has immunosuppressive properties is an inductor of autophagy and/or disrupts homeostatic mTOR feedback loops deserve to be better exploited.

## 4. The Role of mTOR in the Kidney

As previously mentioned, mTOR plays a major role in the regulation of cell proliferation and growth, mainly acting as a metabolic sensor, while low cellular energy supply suppresses mTOR activation and high metabolic input fuels mTOR activation. Whereas, the precise roles played by mTORC1 and mTORC2 complexes in the different types of renal cells is not fully unveiled during development nor in the adulthood, it is suggested that mTOR signaling pathways impact glomerular and tubulointerstitial renal physiological processes [54].

The same also holds truth under conditions of kidney injury. Podocytes, the most vulnerable elements of all kidneys, can adapt to stressful conditions (e.g., metabolic, immunological, and toxic) acquiring a hypertrophic phenotype [55, 56]. Noteworthy, this compensatory mechanism related to size control seems to be mTOR-mediated [57, 58]. In fact, features like podocyte damage and proteinuria are observed in both animal models and transplanted patients upon rapamycin treatment, strengthening the concept that mTOR activity is paramount for adaptive compensatory mechanisms in response to glomerular insult [59–63]. Moreover, studies using genetic models revealed that besides mTORC1 complex, mTORC2 and its downstream target Akt2 also play a role in renal glomerular functions, including podocyte stress surveillance and survival of remaining podocytes in conditions of nephron mass reduction [58, 64]. Furthermore, prevention of mTORC2-Akt2 activation by rapamycin in biopsy tissue from kidney transplant patients was accompanied by increased glomerular apoptosis [64], reinforcing the notion that mTORC2 (along with mTORC1) might contribute to rapamycin-induced proteinuria.

Regarding kidney tubules, much less is known concerning the physiological (and pathological) role of mTORC1 and mTORC2. Apart from proteinuria, subjects under sirolimus therapy may develop hypophosphatemia and hypokalemia; since phosphaturia is a reliable outcome, and considering that *in vivo* mTORC1 inhibition does not seem to affect the apical phosphate reabsorption machinery [65], it could be conjectured whether mTORC1 could affect the basolateral efflux pathways in proximal tubular cells or other unknown hormonal components of phosphate homeostasis. Further research, namely, using mTORC1 ablation in the proximal tubule, is advisory to clarify the precise mechanisms. Concurrently, *in vitro* data have been suggested on the involvement of mTORC2 in renal tubular Na^+^ balance regulation [66]. This hypothesizes, if further confirmed *in vivo*, might be important for some clinically relevant conditions, such as salt-sensitive hypertension or volume overload occurring with congestive heart failure.

## 5. mTOR Inhibition and Renal Diseases

### 5.1. Kidney Transplantation

The true challenge of transplantation research, in addition to the specific advances in surgery, was to improve knowledge about the complexity of the immune system and to design and synthesize drugs able to counteract acute rejection. In the early 1950s, even without effective solutions to prevent rejection, the first successful kidney transplant between genetically related donors was performed, thereby minimizing the role of HLA system, which would ultimately be discovered in 1958. In fact, in 1954, the kidney transplant performed by the Boston group between identical twins was crowned by huge success, with the kidney receptor surviving eight years posttransplant. However, it was imperative to extend the transplantation to unrelated living donors and deceased donors; however, in those cases, the incidence of rejection, with consequent organ loss, remained very high. With the discovery of the first calcineurin inhibitor and its use in clinical practice in 1983, a new era dawned for graft and patient survival. Later, in 1994, a more potent calcineurin inhibitor came into use: tacrolimus. Tacrolimus, in combination with mycophenolate mofetil or mycophenolate sodium (MMF/MPA), showed a remarkable impact on the incidence of acute rejection, which declined to around 5% and 15%, respectively, with a significant improvement in graft and patient survival to over 90% in the first year after transplantation [67].

This advance notwithstanding intensive experience with calcineurin inhibitors has progressively shown their “dark side”, with side effects frequently related to high drug blood concentrations. Adverse effects such as acute and chronic nephrotoxicity, worsening risk of cardiovascular disease, new onset diabetes after transplantation, increased incidence of neoplasms, and viral infections such as CMV, BKV, and oncogenic viruses have been and still are the Achilles' heel of these drugs, and even today continue to fill discussion forums. At the heart of the controversy remains the permanent search for the balance between receiving adequate immunosuppression to prevent graft rejection and minimizing adverse effects, especially nephrotoxicity and cardiovascular events, which continue to be the main cause of death.

Despite all the undisputable therapeutic progress, improvement in long-term graft survival remains lacking. Several factors have been identified to this end, namely, graft quality (older donors, and/or with expanded criteria) and alloantibody-mediated chronic rejection [68]. This multifactorial problem stimulated research on new drugs, alternatives to calcineurin inhibitors, and/or novel immunosuppression strategies which could simultaneously provide two key transplantation objectives: a better long-term graft survival and fewer toxic and adverse effects on the graft and receptor.

The use of mTOR inhibitors in kidney transplantation started in 1990 with the discovery of rapamycin (sirolimus). Exciting results were observed when sirolimus was combined with cyclosporine and prednisone, leading to a significant reduction in the incidence of acute rejection when compared to azathioprine or placebo, despite persistent high triglyceride levels [69, 70]. It was readily observed that the use of these new immunosuppressant drugs could be an attractive alternative to the calcineurin inhibitors, and thus two immunosuppressive strategies were proposed: either the use of mTOR inhibitors without calcineurin inhibitors or maintenance of calcineurin inhibitors in the early posttransplant period with a switch to mTOR inhibitors shortly thereafter (early conversion). The exclusion of calcineurin inhibitors was tested in some studies [71], but with disappointing results due to the high number of acute rejection episodes. Only one center achieved satisfactory results when comparing sirolimus and IL2R antibody induction, calcineurin inhibitor-based regimen [72]. Induction with lymphocyte-depleting antibodies in two therapeutic strategies comparing sirolimus with cyclosporine also showed no advantage, and there was no difference in graft and receptor survival in the first year. This showed that immunosuppression without calcineurin inhibitors was not a good alternative and suggested that sirolimus alone was less potent in controlling the immune response in the early posttransplant period. This disadvantage was not resolved by increasing the dose, a strategy which was associated with more adverse effects [73].

Considering early conversion to mTOR inhibitors, a study on conversion from cyclosporine A (CsA) to sirolimus at three months posttransplantation, combined with MMF and oral steroids, showed that eGFR in the first year was significantly higher in the sirolimus group (68.9 vs. 64.4 mL/min), with no statistically significant difference regarding receptor and graft survival [74]. The incidence of acute rejection occurred mainly after the suspension of corticosteroids, but the difference was not statistically significant. It should be noted that the sirolimus group had higher serum triglycerides as well as more cases of diarrhoea, aphthous ulcers, and acne. Another randomized study with early conversion of cyclosporine to a different mTOR inhibitor—everolimus—while maintaining mycophenolate mofetil (MMF) observed that the everolimus group showed a significant improvement in eGFR (71.8 vs. 61.9 mL/min). These patients, however, also had a higher incidence of biopsy-proven acute rejection (10 vs. 3%) [75]. The follow-up of these patients at five years confirmed that in the first year posttransplant grafts presented better function, but also a higher incidence of acute rejection [76]. Another study following the same line of research found a better eGFR in the sirolimus group compared to the calcineurin inhibitor at one-year posttransplantation, but after two years this difference disappeared [77]. Biopsy-proven acute rejection biopsy was similar in both groups, but the number of deaths was higher in the calcineurin inhibitor group. In both studies, however, a higher incidence of adverse effects was observed, leading to the discontinuation of the mTOR inhibitor.

In the ZEUS study, designed to analyze the incidence of anti-HLA antibodies (specific to the donor), we found significantly higher levels in patients undergoing mTOR inhibitors. It is unknown whether this effect was drug-related or due to corticosteroid suspension [78].

Faced with somewhat disappointing results from the isolated use of mTOR inhibitors and admitting the undisputed superiority of calcineurin inhibitors (CNI) in the control of rejection, researchers sought to explore the complementarity of both drugs, while minimizing their drawbacks and enhancing their advantages. It should be noted that the combination of mTOR inhibitors and CsA had previously been tested in the 1990s, with a low incidence of acute rejection at the expense of a large number of adverse reactions, mainly related to the high doses that were practiced at the time [79]. Sirolimus doses should vary according to the type of calcineurin inhibitor. In fact, the combined administration of sirolimus with cyclosporine increases its toxicity, implying that a lower dose should be used than with tacrolimus [80]. For these reasons, clinical trials started testing the combination of mTOR inhibitors and calcineurin inhibitors using lower doses. In the work by Langer et al., the combination of everolimus (whole blood concentration target blood level > 3 ng/mL) with very low dose tacrolimus (target blood level 2–4 ng/mL) resulted in a low incidence of acute rejection episodes, without compromising graft function [81]. The combination of sirolimus with a reduced exposure to tacrolimus also showed a low incidence of acute rejection and a trend towards better graft function [82]. A meta-analysis focusing on this topic concluded that the association of mTOR inhibitors with low-dose tacrolimus effectively preserves graft function without a significant impact on patient survival and graft rejection when compared to the standard dose of tacrolimus [83]. The most frequently found adverse events in patients were dyslipidaemia and new-onset diabetes after transplantation (about 60 and 38%, respectively), followed by surgical wound complications and hypertension. In accordance with current experience, the combination of mTOR inhibitors with tacrolimus in low dose appears to be a very potent immunosuppressive regimen, considering that the former adverse effects are dose-dependent.

The challenge of combining efficacy and safety while preventing episodes of acute rejection, and maintaining good long-term graft function, is well present in the ongoing TRANSFORM trial (Advancing renal TRANSplant efficacy Outcomes with an eveRoliMus-based regimen) (NCT01950819), whose final conclusions are expected in 2018 [84]. In this trial, the mTOR inhibitor everolimus combined with a low-dose calcineurin inhibitor is compared to mycophenolate with standard CNI exposure, and the long-term effects are observed. The significant number of patients enrolled and three-year follow-up period makes this the largest randomized study ever undertaken in kidney transplantation and is expected to clarify the advantages or disadvantages of utilizing the combined strategy. In the preliminary results published at 12 months, eGFR was similar in both arms [85], and the study also met its key secondary endpoint showing noninferiority with respect to the composite endpoint of tBPAR, graft loss, and death [85]. A decrease in the incidence of viral infection by cytomegalovirus (3.5 vs. 12.5%) and BK virus (3.9 vs. 7.2%) was observed [85]. The preliminary analysis was able to demonstrate the noninferiority of this therapeutic regimen, with the advantage of a lower incidence of viral infections.

According to current knowledge, it is possible to conclude that mTOR inhibitors in kidney transplantation may be satisfactory and effective when applied in the following two strategies: in combination with low-dose calcineurin inhibitors or in early conversion that provided patients with moderate-to-high immunological risk are excluded.

### 5.2. Polycystic Kidney Disease

Polycystic kidney disease (PKD) is a clinically and genetically heterogeneous group of monogenic disorders. This pathologic entity comprises several Mendelian diseases including autosomal dominant polycystic kidney disease (ADPKD), autosomal recessive polycystic kidney disease (ARPKD), and atypical PKD forms [86, 87]. ADPKD is the most common life-threatening hereditary renal disease, with an incidence of 1 : 400 to 1 : 1000 individuals [88]. Disease severity is highly variable, displaying distinct phenotypes ranging from manifestations *in utero* or during infancy (very early onset (VEO) disease) to clinically silent disease well into the second or third decade of life [89, 90]. In contrast, ARPKD typically presents much earlier (1 : 20000 live births among Caucasians). With advancing clinical course, ARPKD pathophysiological features often resemble the pattern of ADPKD, even though a more severe phenotype is often observed [87].

ADPKD is a chronic entity characterized by the appearance of cysts in both kidneys, which may also occur in other organs such as the liver, ovary, pancreas, spleen, and the central nervous system [91]. It is the most frequent hereditary kidney disease that progresses to end-stage kidney disease by the 5th or 6th decade of life, reaching a prevalence of around 8–10% in patients on dialysis [91]. Kidney size can reach significant dimensions as a consequence of the progressive increase in the volume of cysts in about 5% to 8% of the nephrons, leading to a gradual decline of renal function [91]. Renal capsule distension and compression of surrounding renal tissue may lead to complications such as hypertension and chronic pain, whereas the accumulation of urine can precipitate parenchymal infection. The CRISP study (Consortium for Radiologic Imaging Studies of Polycystic Kidney Disease) showed that renal volume and cysts increase at an exponential rate of about 5% per year and that this increase, as detected by magnetic resonance imaging, is accompanied by progressive deterioration in renal function [92].

About 85% of ADPKD is caused by mutations in the PKD1 gene which encodes polycystin-1, a large glycosylated integral membrane protein receptor present in the plasma membrane and in the renal tubular epithelium as well as in the bile and pancreatic ducts [93]. The remaining 15% are the result of mutations in the PKD2 gene encoding polycystin-2 [94]. Polycystin-1 is an adhesion molecule thought to be involved in cell-cell and cell-matrix interactions, whereas polycystin-2 is similar to a voltage-gated calcium channel. Both interact to regulate calcium influx [95]. Mechanisms of cystogenesis are not fully understood, but disruption of ciliary structure and changes in the cyclic AMP (cAMP) secondary to changes in intracellular calcium are responsible for cell proliferation, fluid secretion, and extracellular matrix composition [91]. These pathophysiological changes are mainly due to the overactivation of EGFR, cAMP, and mTOR pathway, leading to great interest in research regarding the inhibition of this signaling pathway in the treatment of this disease [96, 97]. Given that ADPKD patients carry deletions in adjacent genes such as PKD1 and tuberous sclerosis complex 2 (TSC2) which are responsible for the polycystin 1 and tuberin proteins, the hypothesis of a common cystogenic pathway has been advanced [98]. In fact, the TSC2 gene is responsible for the modulation or inactivation of the cell growth signals and proliferation promoted by serine-threonine kinase mTOR, which is abnormally activated in the cystic epithelium of patients with ADPKD. Polycystin 1 inhibits mTOR signaling through its interaction with tuberin. In the absence of this regulatory function, hyperactivity of the mTOR pathway results in a translational increase of the protein through the phosphorylation of S6K and 4EBP1, leading to proliferation, cell growth, and progression of cystogenesis [98, 99].

**Figure 2:**
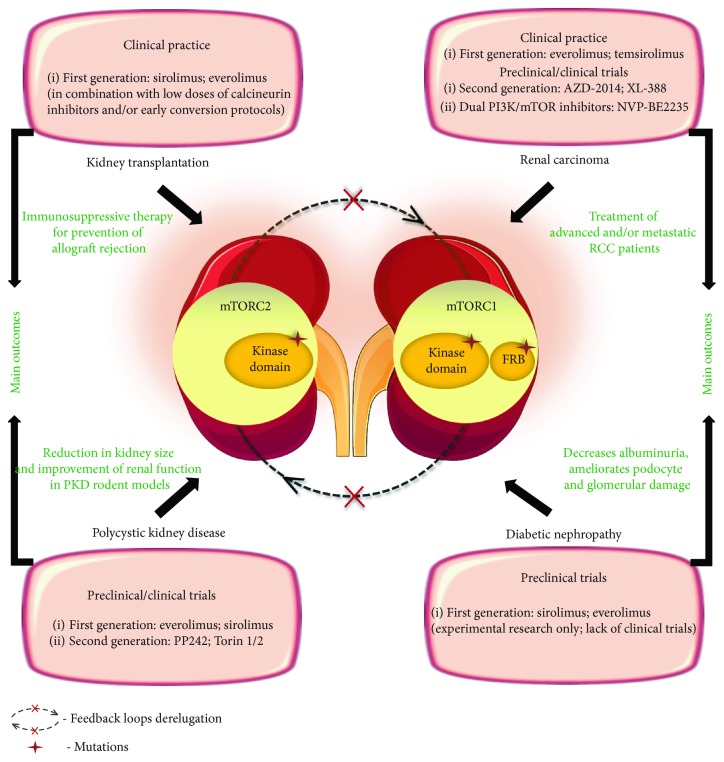
Pharmacological inhibition of mTOR network in renal diseases. A plethora of evidence highlights mTORC1 and/or mTORC2 hyperactivation through deregulation of feedback mechanisms that constitutively regulate mTOR network as well as acquired mutations on mTOR key components, as exemplified in the figure. Herein, it is summarized distinct classes of mTOR inhibitors that are currently available in clinical practice and/or in R&D trial stages in four classes of renal diseases: kidney transplantation, polycystic kidney disease, renal carcinoma, and diabetic nephropathy. The main outcomes from mTOR inhibition are highlighted with green color. Elements of the scheme were drawn using the website https://smart.servier.com/.

Initial studies conducted in preclinical models aimed at establishing whether mTOR inhibition through rapamycin or everolimus (first generation of mTORC1 inhibitors) could ameliorate PKD (see Figure 2). The majority of these studies have reported that these agents elicited a long-lasting reduction in kidney size and an improvement of renal function in rodent models of ADPKD, late-stage nephronophthisis, and models that are not orthologous to any known human mutation [98, 100–104]. Nevertheless, a lack of efficacy was observed in the PCK rat model of ARPKD, Han:SPRD female rats, and early-stage nephronophthisis pcy mice [97, 101, 105]. Rodent models limitations along with a more prominent role of mTOR activity in later phases of the disease were possible explanations suggested by former authors. In light of these studies, mTOR activity inhibition has shown promising results as a therapy to retard the PDK course [104]. Advances in animal models have been recently established in the PKD field. One example is the Vil-Cre;Pkd2^f3/f3^ mice, a ADPKD standardized model showing an important temporal cystic phenotype similar to what occurs in human ADPKD. Interestingly, this new preclinical tool has provided new insights into translational medicine, corroborating the involvement of mTOR pathway (mTORC1–CDK1/cyclin axis) in ADPKD pathophysiology and the efficacy of rapamycin treatment protocols in the improvement of mice survival, cystic phenotype, and renal function [106]. Finally, it is important to emphasize that both mTORC1 (rapamycin-sensitive) and mTORC2 (rapamycin-insensitive) complexes are hyperactivated in PKD [97, 98, 107]. Hence, the use of mTOR kinase inhibitors (that target both mTORC1 and mTORC2) have been hypothesized as a promising strategy to slow cystic kidneys proliferation and improve kidney function. Interestingly, a high-throughput phenotypic screening of kinase inhibitors showed a potent inhibitory activity in cyst size inhibition for most mTOR inhibitors, and a most notable profile was found for Torins 1 and 2 [108]. Additionally, a preclinical study using the Cy/+ rat model of ADPKD highlighted that PP242, another mTOR kinase inhibitor, is able to slow cyst growth and improve kidney function [109]. The influence of mTOR tissue concentration on cyst volume was also evaluated by Novalic et al. which conducted an animal model study using low (3 ng/mL) vs. high (30–60 ng/mL) sirolimus concentrations at different stages of the disease. Only the high-dose group, at the early stage, showed histologically proven inhibition of cystogenesis and regression of cysts, pointing out that effective mTOR inhibition leads to a delay in cyst development and renal volume stabilization, but require higher doses and longer exposure to the drug [110]. Overall, an abundance of preclinical evidence suggests that mTOR inhibitors effectively slow cyst growth, even though the specific role of mTOR complexes is still poorly understood [104].

Because inhibition of mechanistic target of rapamycin (mTOR) effectively slows cyst growth expansion and preserves kidney function in PKD preclinical models, the next logical step was to test the effects of mTOR inhibitors (currently in clinical use as immunosuppressants) on cyst growth in human clinical trials. However, results from large randomized clinical trials testing both sirolimus and everolimus in ADPKD patients are still controversial. In the human randomized study conducted by Serra et al., and after 18 months of observation, patients with eGFR > 70 mL/min and kidney volume of about 1000 mL, rapamycin did not modify the eGFR, nor the total renal volume, compared to the control group, while albuminuria increased in the treated group [111]. In another study, higher doses of sirolimus seemed to stabilize cyst volume, comparing to the conventional therapy-treated patients [112]. The evaluation of the effects of another mTOR inhibitor-everolimus on ADPKD was also performed in a 2-year study that included placebo controls, but the treated group consisted of patients at an advanced disease (stage II or III), and an average kidney volume greater than 1500 mL. It was observed that in treated patients, cysts volume growth rate and renal parenchyma decreased; however, at the end of the study, no eGFR significant difference was found [113]. Stallone et al. also conducted a prospective and randomized study to evaluate the effects of rapamycin on type 1 ADPKD. Patients with eGFR between 40 and 80 mL/min/1.73 m^2^ were divided into three groups receiving ramipril. In two of those groups, a low dose of rapamycin (through levels of 2–4 ng/mL) and a high dose (through levels of 6–8 ng/mL) were given. At 24 months, the authors did not observe any significant difference between treated patients, either in total kidney volume, cystic volume, or estimated creatinine clearance, and it was found that patients receiving rapamycin showed increased urinary protein excretion [114].

Overall, these clinical results were largely disappointing, taking into account the promising effects of mTOR inhibition in PKD animal models and retrospective studies of kidney transplant recipients undergoing immunosuppression with mTOR inhibitors who displayed reduced liver cystic phenotype [115, 116]. Some hypothesis has been figured out to explain such discouraging clinical results. Divergent approaches in terms of sample acquirements, use of different mTOR inhibitors/doses and biomarkers evaluation between experimental groups, may help to explain the lack of clinical efficacy of this class of drugs. In fact, kidney volumes have been extensively used as a surrogate endpoint of disease progression. However, therapeutic strategies that halt kidney enlargement does not necessarily improve renal function, and this is particularly relevant in ADPKD patients who constitutively display enlarged kidneys, even though the renal function is maintained for many years. Hence, from the clinical viewpoint, more adequate biomarkers to assess the efficacy of mTOR inhibition in ADPKD have been proposed, namely, the measurement of changes in GFR, serum creatinine level, and the urinary protein : creatinine ratio [116–118]. Another important feature may rely on the fact that mTOR inhibitors used in these trials may exhibit inadequate tissue penetration at clinically tolerable doses [119, 120]. In this regard, the mTOR kinase inhibitors appear to have a low side effect profile besides their ability to inhibit both mTORC1 and 2 complexes [109, 121]. Taken together, and until now, the results of mTOR inhibition therapy in ADPKD in humans, contrary to the impression left by animal model studies, does not consistently confirm the beneficial impact on renal volume or function. On the other hand, the high dose required to show some efficacy increases the adverse effects incidence, namely, the increase in urinary protein excretion.

### 5.3. Renal Carcinomas

Renal cell carcinoma (RCC) accounts for 2 to 3% of all adult malignancies and is the most common type of kidney cancer [89]. It develops from the proximal tubular cells and is histologically classified as clear cell RCC (ccRCC, ~85%) and nonclear cell RCC (nccRCC, ~15%). ccRCC is frequently associated with the von Hippel-Lindau (VHL) tumor suppressor mutational loss of function and subsequent accumulation of hypoxia-inducible factor (HIF) proteins, leading to the aberrant activation of HIF target genes that regulate angiogenic factors (vascular endothelial growth factor A, epidermal growth factor receptor type 1, platelet-derived growth factor B chain, and transforming growth factor), glycolysis, and apoptosis [122]. Yet, other driver mutations are also involved in the ccRCC development, including those responsible for the constitutive increase in mTOR activation [123, 124]. For example, loss-of-function mutations of PTEN, a negative regulator of mTOR through the PI3K/Akt pathway, are found in nearly 5% of RCC patients. Moreover, loss-of-function mutations of TSC1/TSC2 genes that lead to the inactivation of TSC—a negative regulator of mTOR—are present in patients with tuberous sclerosis, a population particularly predisposed to the development of RCC [125, 126].

RCC is a highly vascularized malignancy and has been relatively resistant to traditional chemotherapy; therefore, the focus of current treatments relies in (i) cytokine-based immunotherapy (e.g., IFN-*α*), (ii) VEGF receptor-associated tyrosine kinase inhibitors (e.g., sorafenib, sunitinib, and axitinib), (iii) anti-VEGF monoclonal antibody, and (iv) mTORC1 inhibitors, taking into account their potential to simultaneously inhibit both tumor cell proliferation and angiogenesis [122, 127]. In fact, mTOR has presented itself as a valid target for the treatment of RCC, and both everolimus and temsirolimus (first generation of mTOR inhibitors) have EMA- and FDA-approved indications for the treatment of RCC particularly in advanced and/or metastatic RCC patients as well as in patients refractory to anti-VEGF therapy (see Figure 2) [128, 129]. Retrospective studies carried out to compare efficacies of everolimus and temsirolimus in mRCC patients suggest that everolimus treatment appears more favourable than temsirolimus, even though prospective trials are needed to confirm these results [122]. The five-year survival of metastatic RCC has been improved after application of mTORC1 inhibitors, even though clinical data is somewhat mixed and the utility of these agents in advanced and/or metastatic RCC (alone or combined with VEGF inhibitors) is currently controversial based on the results from more recent clinical trials (e.g., METEOR and Checkmate 025) [127, 129–132]. These observations are aligned with the poor efficacy of rapalogues in other pathological conditions as they only partially block mTOR signaling. Furthermore, incomplete inhibition of mTORC1 often induces feedback activation of procancerous signaling cascades (e.g., PI3K/Akt and ERK/MAPK).

Recent research efforts have been placed in other classes of mTOR inhibitors [128, 133]. Cho and colleagues tested the antitumor efficacy of NVP-BEZ235, a dual PI3K/mTOR inhibitor, alone or in combination with sorafenib in renal cancer xenografts. The combined protocol showed positive results with enhanced apoptosis and reduction of renal cancer cell proliferation [134]. Another preclinical study focused on AZD2014, a dual mTORC1/2 inhibitor, showed higher *in vitro* efficiency in the inhibition of RCC cell survival and growth as well as RCC cell apoptosis when compared with conventional mTORC1 inhibitors (rapamycin), providing evidence for clinical trials using AZD2014 in RCC treatment [135]. Nevertheless, a randomized phase II study of AZD2014 versus everolimus in anti-VEGF-refractory metastatic RCC showed inferior progression-free survival (primary endpoint) and overall survival with this TOR-KI, despite favourable toxicity and pharmacokinetic profiles (secondary endpoints) [136]. More recently, a novel, selective, and orally available mTOR-KI—XL388—was found to inhibit the survival and proliferation of both established and primary human RCC cells. XL388 was significantly more potent in RCC cell death than rapalogues and showed efficacy in 786-0 RCC tumor growth in nude mice. Moreover, this molecule was also able to elicit HIF-1*α*/2*α* downregulation in RCC cells with putative antiangiogenic effects, strengthening the value of XL388 for future clinic evaluation [137]. Overall, future studies are needed to translate new evidence from basic research into novel multitargeted agents of mTOR network modulation within RCC.

### 5.4. Diabetic Nephropathy

Diabetic nephropathy (DN) is a common complication of type 1 and type 2 diabetes mellitus and is the leading cause of end-stage renal disease (ESRD) worldwide. Clinically, DN is characterized by gradually worsening of albuminuria and GFR decline, in a process that seems to start by glomerular podocyte damage and loss, then progressing to fibrosis of renal glomerulus and of tubulointerstitial region cells. All kidney cell types, including podocytes and mesangial, endothelial, and tubulointerstitial cells, are affected. In opposition to the thesis that DN progression is mainly caused by glomerular protein leakage, it is currently accepted that the glomerular filtration barrier and the tubulointerstitial compartment are an entire dynamic unit that participates in disease evolution.

mTOR pathway signaling abnormalities seem to be present in all the key steps of DN progression, including (i) podocyte damage and loss, an early event in DN that further causes glomerulosclerosis; (ii) overactivation of mesangial cells that promotes increased ECM synthesis and decreased degradation of damaged podocytes; (iii) glomerular endothelial cells and mesangial cell crosstalk that precedes glomerulosclerosis; and (iv) fibrosis and epithelial-to-mesenchymal transition in tubulointerstitial cells [11]. Although the precise mechanisms remain to be clarified, accumulating experimental and clinical evidence supports a major role of mTOR pathway disturbances in DN progression.

Collectively, diabetes is closely linked with conditions that cause mTOR activation, namely, excessive caloric intake, even when preceding obesity, insulin resistance, and overt hyperglycemia development. Activation of mTOR complexes 1 and 2 promotes fat deposition in the adipose tissue [138, 139], which is in agreement with the rapamycin-induced hyperlipidemia seen clinical practice [140]. In conditions of overt diabetes, hyperglycemia further exacerbates mTORC1 activation due to inhibition of AMPK phosphorylation [141]. Concerning the kidney tissue, mTOR activation by diabetic conditions is related to both glomerular and tubulointerstitial changes of DN. Podocyte hypertrophy is a pivotal and early step in the glomerular hypertrophy that precedes proteinuria development and irreversible structural changes, culminating in glomerulosclerosis and nephron loss in DN [142]. Importantly, accumulating evidence from animal models of DN has suggested that mTORC1, via S6K1, participates in such process of renal hypertrophy. The role played by mTOR in podocyte function in conditions of DN was better clarified by the results of two experimental studies based on podocyte-specific genetic deletion of critical components of the mTOR signaling pathway [58, 143]. Briefly, these studies make use of two distinct models to show that mTORC1 overactivation in nondiabetic mice caused a glomerular disease closely resembling DN, while podocyte-specific inhibition of mTORC1 activity protected mice from DN development [58, 143]. Altogether, these studies strongly supported the idea that mTORC1 inhibition could be an effective therapeutic strategy against DN development. A drawback of this approach was the development of proteinuria when raptor expression was ablated in podocytes, which is in line with the known proteinuric effect of rapamycin treatment in both animal models and humans [59–63]. Other relevant metabolic side effects of rapamycin should be also noticed at this point, including hyperglycemia, insulin resistance, and dyslipidemia, which seem to be related to glucose and lipids metabolism in the pancreas and in the peripheral insulin resistant tissues (liver, adipocyte tissue, and muscle), as previously reported in animal and human studies, some of them from our own group [37, 144–150].

Apart from impaired mTOR signaling in podocytes that contributes to podocyte loss, mTORC1 activation seems to be associated with renal hypertrophy and matrix expansion, overexpression of type IV collagen, fibronectin, and laminin [11]. mTOR inhibition by rapamycin prevents these effects and ameliorates the key glomerular changes found in DN, such as hypertrophy, basement membrane thickening, and mesangial matrix accumulation, accompanied by a decrease in albuminuria [142, 151]. Regarding interstitial fibrosis, mTOR seems to be able to stimulate fibroblasts proliferation, collagen synthesis, and expression of profibrotic cytokines, such as TGF-*β*1 and CTGF, which are pivotal players in the tubulointerstitial damage, a crucial feature of DN [142, 151]. Finally, mTOR seems also to participate in the epithelial-to-mesenchymal transition, a mechanism that is inhibited by rapamycin [142, 152].

To conclude, accumulating evidence, mostly from animal models, shows that mTOR activation might have a role on DN progression by acting on different kidney cell types and mechanisms, suggesting that mTOR inhibition could be, in theory, an attractive therapeutic strategy to overcome DN. However, the recognition of relevant side-effects in transplanted patients treated with rapamycin, such as hyperglycemia, insulin resistance, and dyslipidemia, may explain the scarceness of preclinical studies and lack of clinical trials using mTOR inhibitors to prevent or modify DN course (see Figure 2).

## 6. Conclusions and Future Directions

The mTOR pathway is an exciting area of research in many biomedical areas of knowledge, including aging, metabolism, neurobiology, oncobiology, and cardiovascular and renal diseases. Regarding the kidney, activation of mTOR complexes (mainly mTORC1) has been recognized to participate in a multiplicity of renal processes underlying the development of glomerular and tubular damage/fibrosis, such as regulation of podocyte size (hypertrophy and/or proliferation), epithelial-to-mesenchymal transition, and tubulointerstitial inflammation.

Inhibition of mTOR using rapamycin (sirolimus) or everolimus (a rapalogue), alongside with other immunosuppressive agents and depending on the immunological risk, has been a well succeeded strategy to improve outcomes in renal transplanted patients, regardless of the possibility of drug-induced proteinuria and other metabolic side-effects, which should be closely monitored and controlled. However, further clinical data is still needed to understand the putative benefits of mTOR inhibitors against the development of certain types of cancers and viral infections in transplanted patients.

Concerning PKD, in particular, the autosomal dominant form (ADPKD), the few clinical data available with mTOR inhibition was unable to confirm the preclinical studies in animal models. Therefore, clinical trials with sirolimus and everolimus have not improved renal volume or function at doses that do not cause significant adverse effects, namely, the increase in urinary protein excretion. Currently, there are not enough data to propose mTOR inhibition in PKD clinical practice. Further disclosure of (i) mechanistic insights of mTOR complexes in PKD pathophysiology, (ii) assessment of more potent and specific mTOR inhibitors, and (iii) careful systematization of clinical trials is paramount to overcome current drawbacks that postpone the translation of mTOR modulation from the benchside to PKD clinical practice.

As regards to RCC, in particular, in advanced and/or metastatic forms, the first generation of mTOR inhibitors (temsirolimus and everolimus) is already in clinical use and has been showing some efficacy, particularly when combined with VEGF modulators. However, the clinical data available remains controversial, namely, due to resistance-acquired phenomena and activation of prooncogenic pathways that limit the long-term use and outcome. Therefore, new pharmacological strategies targeting the mTOR network are currently under preclinical evaluation, which is focused on mTOR-KIs and dual PI3K/mTOR inhibitors.

Regarding the possibility of using mTOR inhibitors to prevent the progression of DN, rapamycin has been shown an ability to ameliorate mesangial expansion, glomerular basement thickening, and release of proinflammatory cytokines or chemokines by monocytes and macrophages. In spite of this amount of promising preclinical data, rapamycin is associated with some metabolic and renal side-effects, namely, insulin resistance and proteinuria, which could compromise its wide-spread use in some conditions. It should be noted that most of the actual knowledge on mTOR pathway in DN was obtained by using pharmacological inhibition of mTORC1 with rapamycin; nevertheless, it has been suggested by studies using animal models that mTORC2 activation also has a role in DN, which should be further exploited.

Although remarkable insights have been achieved over the last years, there is an ample room to improve our knowledge regarding the roles played by mTOR complexes and pathways in kidney physiology and pathogenesis of several renal diseases. In particular, further studies are required to disclose the precise mechanisms underlying the glomerular and tubulointerstitial actions of mTORC1 and mTORC2 in order to improve management of renal diseases and to reduce glomerular side effects and proteinuria reported with the traditional mTOR inhibitors currently available. Further insights are also still needed concerning the upstream regulation of mTOR, the identification of downstream mTOR targets, and, importantly, the specific role played by the regulatory proteins that interact with mTOR in both mTORC1 and mTORC2 complexes, such as Deptor, in order to unveil the impact of the mTORC1-mTORC2 interactome. Likewise, further research, particularly in the clinical setting, is required regarding the impact of mTOR inhibition in immune cells and the ability to ameliorate age-related cellular decline. Finally, the insights hopefully coming in the near future from the studies ongoing with new pharmacological approaches targeting the intricate mTOR network, such as dual PI3K/mTOR inhibitors and new-generation inhibitors (namely, mTOR-KIs), might be able to open new avenues in the treatment of renal diseases in which the impaired mTOR pathway plays a relevant pathological role.
